# American Society of Clinical Oncology–Sponsored Oncology Student Interest Groups in Latin America

**DOI:** 10.1200/GO.20.00363

**Published:** 2020-09-30

**Authors:** Andrea Anampa-Guzmán, Andrea Denise Brito-Hijar, Cristian Antonio Gutierrez-Narvaez, Anthony Raul Molina-Ruiz, Victor Simo-Mendoza, Miguel González-Woge, Andrea de la O Murillo, Roberto Leon-Ferre

**Affiliations:** ^1^ Faculty of Medicine, Universidad Nacional Mayor de San Marcos, Lima, Peru; ^2^ Sociedad Cientifica de San Fernando, Lima, Peru; ^3^ Faculty of Medicine, Universidad Autónoma de Baja California, Mexicali, Mexico; ^4^ Faculty of Medicine, Universidad Anáhuac, Naucalpan de Juárez, Mexico; ^5^ Faculty of Medicine, Universidad Autónoma de Coahuila, Coahuila, Mexico; ^6^ Division of Medical Oncology, Department of Oncology, Mayo Clinic, Rochester, MN

## Abstract

**PURPOSE:**

To describe the characteristics of the members of the ASCO-sponsored Oncology Student Interest Groups (OSIGs) in Latin America.

**METHODS:**

This was a multicenter cross-sectional study. We surveyed 97 OSIG members from three medical schools in Peru and Mexico. We administered a 60-question survey covering topics including personal background, oncology training experience, and professional practice expectations and preferences.

**RESULTS:**

A little more than one half of the surveyed OSIG members were female. More than one half had a visa to visit the United States and had an advanced level of English. One half of the OSIG members were also ASCO members. Most participants agreed or strongly agreed that participation in their OSIG increased their interest in cancer-related specialties (94%) and provided professional networking opportunities (94%) and that it was accessible to all students (91%). Most participants believed that their OSIG had sufficient resources to carry out its activities. Students were asked to rate their interest when they entered medical school versus at the time of the survey. Most of the members were strongly interested in pursuing surgical oncology. The majority of members were somewhat interested or very interested in palliative care and medical oncology.

**CONCLUSION:**

To our knowledge, this is the first study that provides data on medical student perceptions of the Latin American OSIGs sponsored by ASCO. Student perceptions of medical oncology and the impact of OSIGs were generally positive. Given the shortages of oncology specialists in Latin American and elsewhere, strategies to engage medical students in the pursuit of cancer-related careers are becoming increasingly essential.

## INTRODUCTION

ASCO is an organization that connects nearly 45,000 oncology professionals around the world. In Latin American countries, where cancer is the second-leading cause of death, there is a shortage of medical oncologists to meet the demands of patients from the region.^1^ Cancer is the third- and first-leading cause of mortality in Mexico and Peru, respectively.^2^^,^^3^ Despite the burden of this disease, there are just 0.48 and two oncologists for every 100,000 people in Mexico and Peru, respectively.^4^ For comparison, the United States has 16.1 oncologists for every 100,000 people.^5^ The ratio of new cancer cases per clinical oncologist is 420 for Mexico, 331 for Peru, and 137 for the United States. In Mexico and Peru, the majority of oncologists work in their respective capital cities, resulting in significant shortages in smaller towns and rural areas.^6^ It is important to note that in Peru and Mexico, the field of medical oncology treats only patients with solid tumors; in the United States, the field of oncology-hematology involves patients with blood diseases as well as those with solid tumors.^7^ Unfortunately, data regarding the practice of other cancer-related specialties in Mexico and Peru, such as radiation oncology and surgical oncology, are not available.

**CONTEXT**

**Key Objective**
What are the characteristics of the members of the ASCO-sponsored Oncology Student Interest Group (OSIG) in Latin America?
**Knowledge Generated**
A little more than one half of the surveyed OSIG members were female. More than one half had an advanced level of English. One half of the OSIG members were also ASCO members. Most participants agreed or strongly agreed that participation in their OSIG increased their interest in cancer-related specialties and provided professional networking opportunities and that it was accessible to all students. Student perceptions of medical oncology and the impact of OSIGs were generally positive.
**Relevance**
Given the shortages of oncology specialists in Latin America, strategies to engage medical students in the pursuit of cancer-related careers are becoming increasingly essential.

The strategic plan of ASCO is to support the global oncology community to reduce the burden of cancer. As part of this plan, ASCO is interested in increasing medical student engagement and interest in cancer-related careers, both within the United States of America and internationally.^8^ Consistent with this, ASCO developed a program to sponsor Oncology Student Interest Groups (OSIGs), which are made up of medical students and residents who share a common interest in the field of oncology and meet regularly to learn more about cancer, patient care, and pursuing a career in oncology.^9^ In this way, ASCO seeks to encourage the pursuit of specialties related to oncology to face the shortage of cancer control specialists.^10,11^ ASCO offers a 2-year sponsorship that includes funding, opportunities to connect with cancer professionals, and the opportunity to attend the annual ASCO meeting. OSIG program benefits include access to situational mentoring and networking opportunities. Moreover, OSIG members have the opportunity to submit research to the abstract forum at the ASCO annual meeting, apply for travel stipends to that meeting, receive complimentary student registration for it, and the opportunity to be paired with an oncology mentor as part of the buddy program. Each OSIG receives funds based on the needs expressed in a detailed budget sent as part of the initial application.

For the period of 2018-2019, at the time of the current study, ASCO sponsored 87 OSIGs across 33 states in the United States and five countries.^10,11^ In Latin America, there were only four (4.6%) of all OSIGs, including one in Peru at the National University of San Marcos (Universidad Nacional Mayor de San Marcos [UNMSM]) and three in Mexico at: (1) the University of Anahuac (Universidad Anáhuac [UA]), (2) the Autonomous University of Baja California (Universidad Autonoma de Baja California [UABC]), and (3) the Autonomous University of Coahuila (Universidad Autonoma de Coahuila [UAC]).^10^ After the completion of this study, for the period of 2019-2020, two additional OSIGs were founded in Latin America, one in Brazil and another in Mexico,^12^ to bring the total number of Latin OSIGs to six (6.74%) of all OSIGs).

Few studies have evaluated the factors that influence the choice to pursue a career in cancer-related specialties. A French study found that among residents, the most decisive motivation for choosing medical oncology was the experience they had had at the undergraduate level. The main reasons listed were the interest of the students in cancer research, the multidisciplinary nature of oncology, and the significant variations in clinical practice and the perception of the lifestyle offered by these specialties.^13^ In a study conducted by the Boston University School of Medicine, the implementation of a Student Oncology Society, a multidisciplinary interest group led by students, was associated with an increase in student interest in oncology compared with when the students started medical school.^14^

In this study, we sought to gain additional understanding of the membership of the ASCO-sponsored OSIGs in Latin America. We surveyed OSIG members in Peru and Mexico to describe the basic demographic characteristics of their members, their experience and perceptions of oncology specialties, their interest in cancer-related careers, and their engagement with ASCO. The study was limited to these two countries, because these were the only two countries in Latin America with ASCO-sponsored OSIGs at the time.

## METHODS

This study was cross-sectional and multicenter. The population consisted of all of the student members of the ASCO-sponsored OSIGs in Latin America during 2019, including at UNMSM in Peru, as well as at UABC, UA, and UAC in Mexico. A brief description of the characteristics of each university and its curriculum can be found in the Data Supplement. We collected data through a 60-item questionnaire that had three sections:

1. Personal background
2. Oncology training
3. Professional practice expectations and preferences.

To ensure clarity for both Peruvian and Mexican students, the survey was developed in Spanish by A.A.-G., A.D.B.-H., C.A.G.-N., A.R.M.-R., and M.G.-W. The survey contained sections regarding sociodemographics, perceptions of specialties related to oncology, training in oncology during medical school, and preferences and expectations for the OSIG at all of the surveyed universities. The research ethics committee of UNMSM approved the protocol (19-0091).

Participants provided informed consent before filling out the survey, which was conducted using Google Forms. For qualitative variables, descriptive statistics were used, including frequencies and percentages. To analyze interest in cancer-related careers, we used a Likert scale ranging from 1 to 10 (1 = no interest, 10 = strong interest). Because of the differences in training duration and curriculum between countries, we asked specific questions only regarding medical oncology as a medical specialty. We assessed the level of interest in only the specialties of surgical oncology, palliative care, and radiation oncology. Students were asked to rate their interest when they started medical school versus at the time of the survey. Data were subjected to a paired *t* test. All analyses were performed in STATA. All *P* values are two sided at a significance level of .05.

## RESULTS

The four surveyed ASCO-sponsored OSIGs consisted of 125 medical students. We sent out a survey to each one of these students, and a total of 97 students responded (response rate, 77.6%). Only one student from the UAC OSIG (n = 15) in Mexico responded, and as such, this OSIG was omitted. A total of 96 surveys were analyzed. The baseline characteristics of the surveyed Latin American OSIG members are listed in Table 1. The majority of OSIG members were female. None of the students surveyed were citizens of the United States, but more than one half had a visa to visit the United States, and 62.5% self-reported having an advanced English level. Most had been a member of their OSIG for < 1 year. Just over one half were also ASCO student members. More than 75% reported having a family member diagnosed with cancer.

**TABLE 1:**
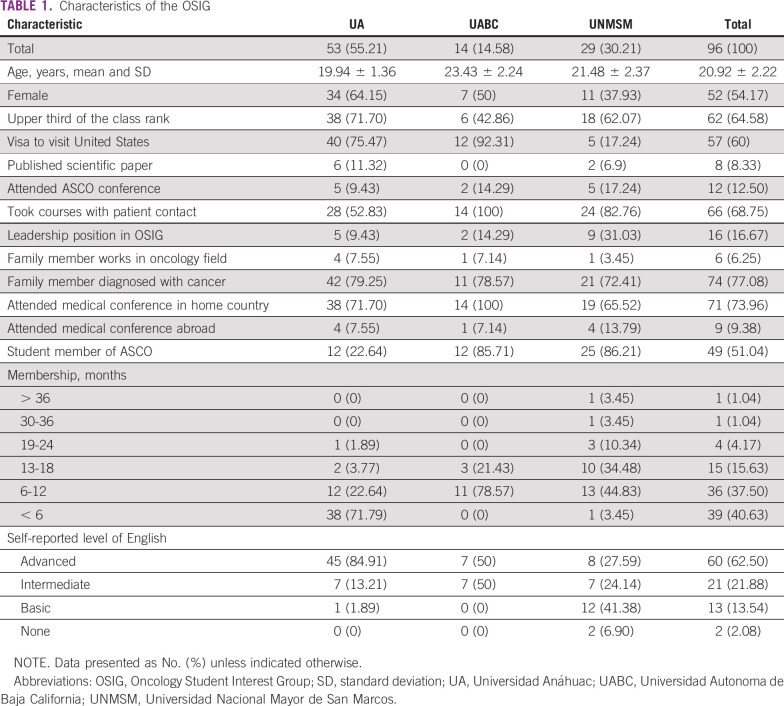
Characteristics of the OSIG

The perceptions of the OSIG student members regarding oncology specialties are listed in Table 2. The majority reported agreement or strong agreement with the phrases “medical oncology is an attractive specialty,” “enjoys an elevated status among other specialties,” and “elevated social status,” “enjoys scientific prestige,” “has an essential role in society,” and “it is interesting for research.” Conversely, 44.79% reported agreement or strong agreement with the statement “medical oncology has a high salary,” and 43.75% of students were neutral about it. One half of the participants agreed or strongly agreed with medical oncology providing a “good work environment.” With regards to oncology education in their medical school, most members agreed or strongly agreed that “there are sufficient reasons to justify its mandatory theoretical-practical learning,” “oncology teaching must be mandatory,” and “the training I receive in oncology seems appropriate to me.” About their respective OSIG, the majority of students agreed or strongly agreed that these organizations increased their interest in oncology, promoted the establishment of professional connections, and were accessible to all students, and that their OSIG had sufficient resources to carry out its activities.

**TABLE 2:**
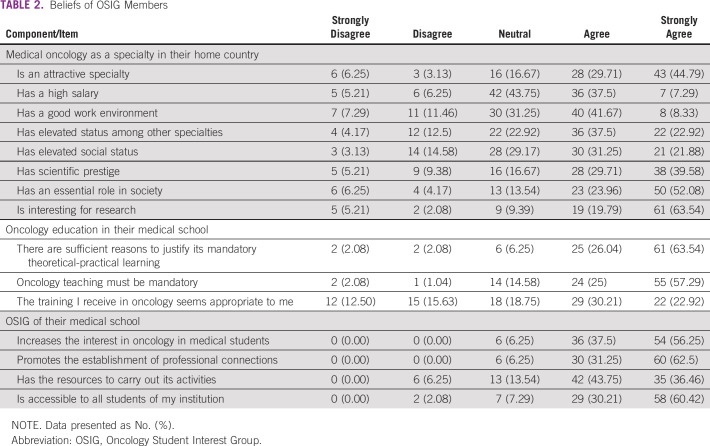
Beliefs of OSIG Members

The factors that influenced the students’ interest in oncology are listed in Table 3. The majority expressed that their interest in oncology was positively affected by personal experiences in medical school, the OSIG, their experience as either a patient or relative of a patient, and the opinions of other physicians and oncology specialists. The views of family and friends had a positive influence, and social media had a variable impact. Members were asked about their interest in oncology as a medical field when they started medical school compared with at the time of the survey. Participants in all groups reported a significant increase in their interest (Data Supplement).

**TABLE 3:**
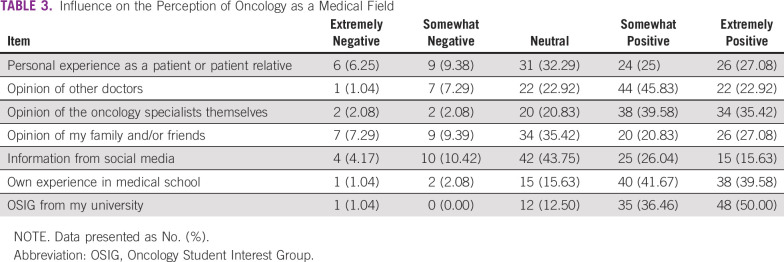
Influence on the Perception of Oncology as a Medical Field

Table 4 lists the interest of OSIG members in careers in oncology. Most of the members were strongly interested in pursuing surgical oncology. The majority of members where somewhat interested or very interested in palliative care and medical oncology. A minority of students reported an interest in radiation oncology.

**TABLE 4:**
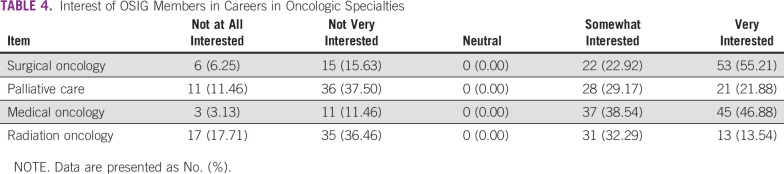
Interest of OSIG Members in Careers in Oncologic Specialties

## DISCUSSION

The perception among the student participants of income potential as a medical oncologist was mixed. In Peru, oncology is the medical specialty with the lowest average wage. According to publicly available information, the average yearly salaries for Peruvian and Mexican medical oncologists are equivalent to $17,662.68 and $11,739 USD, respectively.^15^ Medical oncologists in Peru are less likely to have higher incomes than physicians without specialty training.^16^ In the United States, a 2011 study on the factors associated with choosing oncology and hematology as a specialty reported that fewer than one half of oncologists considered salary as a determining factor for their career choice.^17^ Whether the same is valid for Latin American students is unknown.

Students considered that the ASCO-sponsored OSIGs had a positive influence on their interest in oncology and provided an opportunity to network with cancer specialists. In 2007, the French Association of Residents in Oncology surveyed every resident in medical oncology in the country to determine the factors that influence the decision to choose oncology for residency. The survey found that 83% consider training as a motivating factor for their choice. In the French study mentioned previously, 24% of participants reported that they did not receive adequate oncologic training during medical school, which is strikingly similar to the 27% of students with a similar perception in our study.^18^ This finding highlights the need to identify opportunities for improvement in the current oncology curriculum of medical schools in Latin America.

A study by Sherwood et al,^19^ at a meeting of Interest Groups in Oncology at the University of Saint John in Canada, found that students perceived interest groups as necessary. Activities such as clinical skill workshops and training in delivering bad news were particularly valued by students. The ability to connect with mentors in oncology was also an influential factor in their future choice. The authors pointed out that sufficient funding was vital to the success of these groups. These findings highlight the importance of initiatives such as the ASCO-sponsored OSIGs in funding for these types of activities within Latin America. The surveyed members perceived that the financing available through ASCO was adequate to maintain their activities.

It is important to note that sponsored OSIGs are the only sponsor initiatives by ASCO that explicitly include medical students outside the United States.^12^ This is especially important because, according to the Accreditation Council of Graduate Medical Education, 36.4% of active hematologist-oncologists are international medical graduates (IMGs).^20^ Moreover, 40.9% of hematology-oncology fellows are IMGs.^21^ Together, these statistics indicate that international physicians are a significant component of the oncologic workforce. Given the fact that the majority of members of ASCO-sponsored OSIGs had an advanced level of English and a visa to visit the United States, the potential exchange of medical students is feasible.

Personal experiences with cancer, experiences in medical school, and the opinions of medical experts and educators positively influence the decision to select oncology as a specialty. One study performed in the United States and Canada found that personal experiences were the most significant factor, with the opinions of physicians exerting a secondary influence.^22^ Another study in Mexico found that medical educators and personal experiences in medical workshops and activities were the most influential factors in the choice of medical specialty.^23^ Conversely, the opinion of family members and friends seemed to have little influence. This is in agreement with two separate studies performed in Saudi Arabia and Brazil, in which students reported that the opinions of family members and friends had little or no significant influence on their choice of medical specialty.^24,25^ The majority of OSIG students agreed that medical oncology is an attractive specialty, is interesting for those interested in research, plays an essential role in society, and enjoys scientific and social prestige. However, we understand that these experiences and feelings can be part of the self-selection elements of those interested in oncology.

It has been reported that students initially interested in oncology lose interest as they advance in their careers.^26^ In our study, we found that OSIG members reported a significant increase in their interest in the field of oncology compared with when they entered medical school. A limitation of this finding is that we asked students to report their interest in oncology when they started medical school and at the current time in the same survey, which introduced the potential for recall bias. In addition, the students surveyed were all active members of the ASCO-sponsored OSIG, and, as such, there was potential for selection bias. To overcome these biases, student specialty interest at the time of medical school enrollment would need to be collected prospectively and assessed at multiple times as the student progresses. Because such information is not currently available in the surveyed medical schools, we consider that it is valuable to report our limited findings. Another limitation is that this survey included only two Latin American countries, and since the completion of this study, other countries have joined the ASCO-sponsored OSIG initiative. As such, the current results may not be representative of the experience across all of Latin America. The number of Latin American OSIGs has been increasing annually; this expansion is important because of the low number of oncologists in these countries. Additional studies characterizing ASCO-sponsored OSIGs globally should be considered to determine the influence of this initiative in different parts of the world.

To our knowledge, this is the first study to provide data about Latin American OSIGs sponsored by ASCO. We expect that our data will help inform the design of future initiatives aimed at engaging medical students in cancer-related careers. Student perceptions of medical oncology and the impact of ASCO-sponsored OSIGs at their institutions were generally positive. Given the shortages of oncology specialists in Latin American and elsewhere, strategies to engage medical students in the pursuit of cancer-related careers are becoming increasingly essential.
